# Peel and Penetration Resistance of Porous Polyethylene Terephthalate Material Produced by CO_2_-Assisted Polymer Compression

**DOI:** 10.3390/molecules24071384

**Published:** 2019-04-09

**Authors:** Takafumi Aizawa

**Affiliations:** Research Institute for Chemical Process Technology, National Institute of Advanced Industrial Science and Technology, 4-2-1 Nigatake, Miyagino-ku, Sendai 983-8551, Japan; t.aizawa@aist.go.jp; Tel.: +81-22-237-5211

**Keywords:** peel resistance, penetration resistance, porous polymer, polyethylene terephthalate, CO_2_-assisted polymer compression, cross-sectional model

## Abstract

CO_2_-assisted polymer compression (CAPC) involves adhering fiber sheets without impurities at room temperature and producing porous materials suitable for use in medical and skin-contactable products. The mechanical strength of the resultant porous material has not yet been reported. The penetration resistance of the CAPC material, which is a laminated material comprising fibrous polymer sheets, was measured, and this increased gradually with the density. Additionally, a T-type peel test was performed on the CAPC material, and the peel resistance increased rapidly with the density. The peel resistance enhancement is effectively explained by the cross-sectional analysis model.

## 1. Introduction

Polymers are used ubiquitously because they are lightweight and strong [1]. For example, polyethylene terephthalate (PET) is widely used for beverage bottles and clothes. In particular, applications of foamed polymers are currently being investigated to further reduce the weight of products and many products have been developed. Polymers are proposed not only to be used alone but also to be complexed with inorganic particles to make them multifunctional [2]. Polymers are often used in medical applications because their absorption in the body is suppressed [3,4]. For such delicate applications, a polymer without impurities is required, necessitating a processing method that involves no adhesives. Additionally, from an environmental viewpoint, solvents and other chemicals should be eliminated as much as possible.

CO_2_, an atmospheric gas with extremely low toxicity, has attracted much attention with respect to its use in environmentally friendly processes [5] and is ideal in the field of pharmaceuticals [6]. CO_2_ is readily soluble in polymers, as shown in reports of a *specific intermolecular interaction* between CO_2_ and polymers [7]. Processes combining CO_2_ and polymers [8], polymer synthesis in CO_2_ [9,10], fine polymer particle production using CO_2_ [11,12], foamed polymer manufacture [13,14], and polymer nanocomposite foam manufacture [15,16] have been proposed.

Recently, a CO_2_-assisted polymer compression (CAPC) method that easily produces porous polymeric materials by compressing fibrous sheets in CO_2_ [17] was proposed. The process involved a simple configured device that does not include a high-pressure pump or temperature controller, thereby reducing the equipment cost. Additionally, only a small amount of CO_2_ was required, which reduced the running cost. Since the process only used CO_2_ without adhesives, it provided an innocuous material without impurities. Materials obtained by this method are porous with through-holes and voids between fibers. Additionally, the porosity can be controlled by changing the operating conditions [18]. Furthermore, a drug-containing sample was prepared, and a controlled sustained drug release was demonstrated [19]. Porous materials are often used as a support to carry drugs and catalysts; however, it is difficult to place these substances in the center of a thick porous material. By using the CAPC method, a porous material carrying drugs or catalysts can be prepared by laminating fiber sheets bearing these substances. Even when the number of stacked sheets was increased, the compression time remained constant, and samples which were several centimeters thick were successfully manufactured. Since the through-holes are open, they facilitate water vapor release, suggesting that the fabricated materials may be sweat-resistant. Moreover, the materials are expected to be resistant to penetration from the direction perpendicular to the fiber sheet because the fibers overlap. Consequently, porous materials prepared by CAPC are expected to be highly suitable for medical support devices such as corsets or protective athletic gear, such as knee and shin pads. However, when this CO_2_-based adhesion method was developed, no stable adhesive strength data could be obtained due to the small sample size; therefore, the adhesive strength could not be quantified. This lack of data precluded the evaluation of whether the fabricated materials were suitable for use in the above applications. However, because peeling and penetration tests can now be reproducibly performed by devising a method of preparing small samples, the relation between the CAPC conditions and the peel and penetration resistance of porous PET materials is reported herein. Additionally, the peel resistance is analyzed by constructing a model. This data will be useful for medical and industrial researchers.

## 2. Results and Discussion

**Figure 1:**
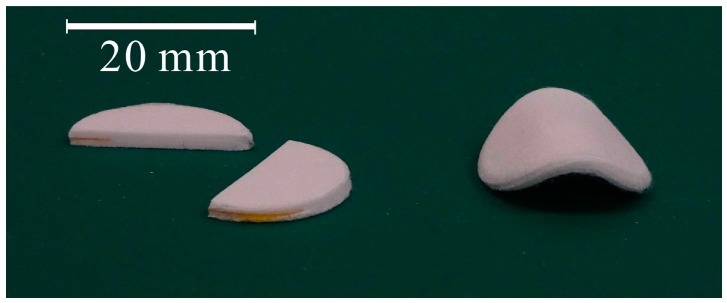
Cut and bent samples. Samples with a density of 0.64 g/mL were cut or bent.

Figure 1 shows cut and bent samples with a density of 0.64 g/mL. The samples were cut and bent without peeling off. In a previous study, samples did not peel off even when immersed in water for over 12 h [19].

**Figure 2:**
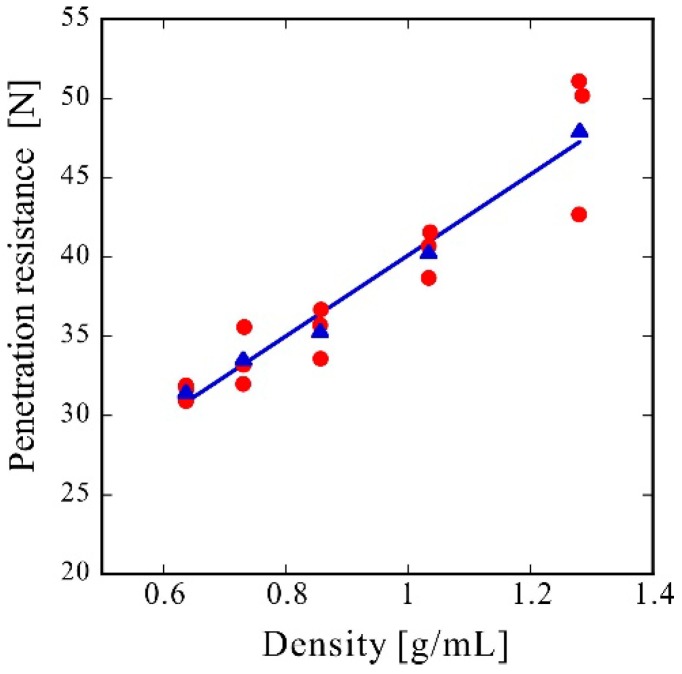
Penetration resistance. Experimental results (**red circle**) and averages of three experimental data points (**blue triangle**) at each bulk density and the linear fitting result (**blue solid line**).

Figure 2 shows the penetration test results. The red circles represent three individual measurements under each condition, and the blue triangles represent their average values. The solid line is the result of the linear fitting of the mean values using the least-squares method. Since the material was a non-woven fabric, the fibers are loosely distributed inside the sheet. Thus, when a penetration test was conducted, the fibers can escape from the needle, and almost no penetration resistance should be observed. The fibers were pressed into each other to obtain a three-dimensional structure, which drastically improved the penetration resistance.

**Figure 3:**
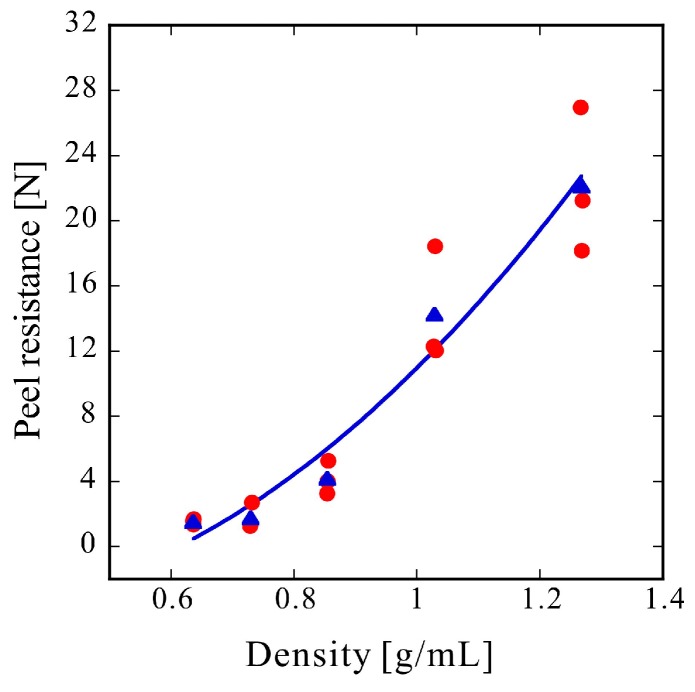
Peel resistance. Experimental results (**red circle**) and averages of three experimental data points (**blue triangle**) at each bulk density and the quadratic fitting result (**blue solid line**).

Figure 3 shows the peel test results. A PET pellet density of 1.34 g/mL, as described in the datasheet, corresponds to a porosity of zero. The red circles represent three individual measurements under each condition and the blue triangles represent their average values. The solid line is a quadratic fitting of the average values using the least-squares method. In comparison with the linear and exponential fitting results, the quadratic fitting result was relatively good. The adhesion principle of CAPC is that bonding points are formed by compressing locations where plasticized fibers overlap [17,18]. Consequently, the adhesive strength should be expressed by the number of bonding points per unit area multiplied by the adhesive strength per bonding point. The number of bonding points per unit area is expected to increase with the bonding strength. Furthermore, the bonding strength per bonding point is also expected to increase with the bonding strength. Since these effects are synergistic, the quadratic fitting was relatively good. However, the type of equation expressed theoretically will be examined in future work, after further experiments with different materials.

The peel resistance rapidly increased with the bulk density increase which was induced by compression. The peel resistance values in terms of 10 mm wide reference samples were 0.5 and 2.0 N for the one-sided (Figure 9c) and two-sided (Figure 9d) adhesive surfaces, respectively. The peel resistance of the sample with a porosity of 53% and a density of 0.64 g/mL was higher than that of the transparent tape with one adhesive surface. Likewise, the peel resistance of a sample with a porosity of 36% and a density of 0.85 g/mL was higher than that of the transparent tape with adhesive surfaces on both sides. Although the required bonding strength depends on the application, the required adhesive strength may be achievable because the adhesive strength rapidly increases with the press strength.

Herein, it was hypothesized that an increase in adhesion strength (peel resistance) would greatly affect the penetration resistance; however, even for the sample with a density of only 0.64 g/mL and a porosity of 53%, the penetration resistance was sufficient. The penetration resistance increased linearly with the density. In comparison with the rapid increase in peel resistance with bulk density, shown in Figure 3, the increase in penetration resistance was gradual (Figure 2), indicating that penetration resistance is attained if fibers can withstand the forces of penetration with a needle. Therefore, only an increase in strength resulting from an increase in density, rather than an increase in adhesion strength, substantially affects the penetration resistance. The reference penetration resistance values of the PE film and EVA foam were 2.3 and 9.2 N, respectively. Therefore, the porous PET materials produced by CAPC herein exhibited high penetration resistance, indicating potential suitability for use in protective gear or clothing in fields requiring safety and security.

**Figure 4:**
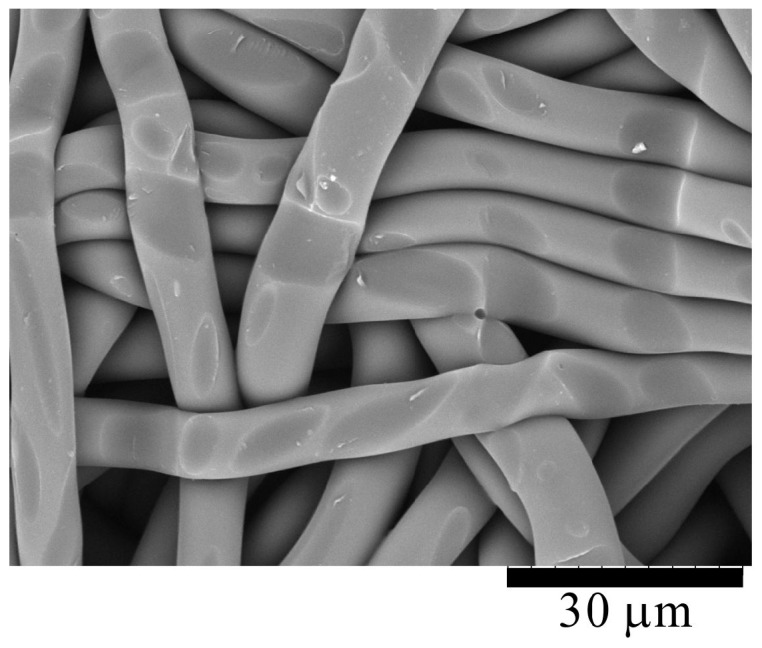
SEM image of an intermediate layer of a CO_2_-assisted polymer compression (CAPC) sample. Traces of fibers pressed on their surfaces are observed.

Figure 4 shows a scanning electron microscope (SEM) image of the intermediate layer surface of a CAPC sample. This image shows that aggregation through point adhesion between fibers is an adhesion mechanism of a CAPC sample. The detailed adhesion mechanism of CAPC has been explained in my previous papers [17,18]; hence, only a brief description is provided herein. In the case of thermoplastic polymers, the polymer’s shape is maintained by the friction of the polymer chains. When the polymer is impregnated with CO_2_, the CO_2_ molecules reduce the friction between the polymer chains, thereby increasing the polymer’s fluidity. By compressing the polymer in a plasticized state, bonding points are formed at the overlapping portions of the fibers. The adhesion of the sample is completed by exhausting CO_2_ and fixing the polymer’s shape.

**Figure 5:**
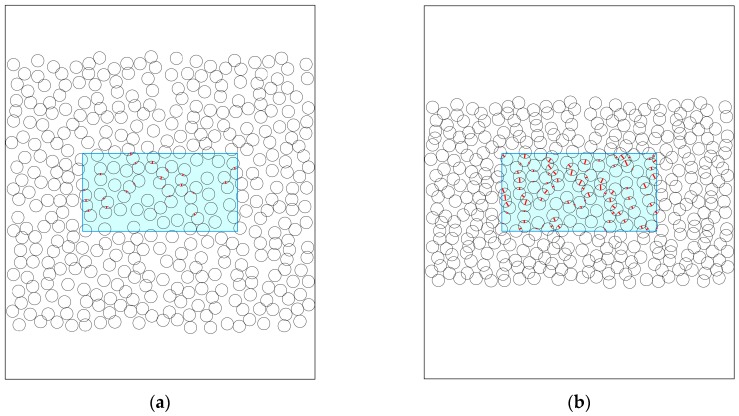
Cross-sectional analysis at compression ratios of (**a**) 0.75 and (**b**) 0.5. The overlap length is shown by red lines.

The increases in the number of bonding points and the bonding strength per bonding point were verified using a cross-sectional analysis model. A porous material should originally be considered as a three-dimensional structure. However, a cross-sectional method is sometimes used to evaluate tortuosity in diffusion analysis [20,21,22]. The results for the initial arrangement shown in Figure 11 are shown in Figure 5 for compression ratios of 0.75 and 0.5. The overlap length, where the overlap center is within the analysis area, is shown by red lines. As shown in Figure 5, as the compression ratio decreased (higher compression), the number of bonding points (the number of red lines) increased, and the overlap length (length of the red line) appeared to increase.

**Figure 6:**
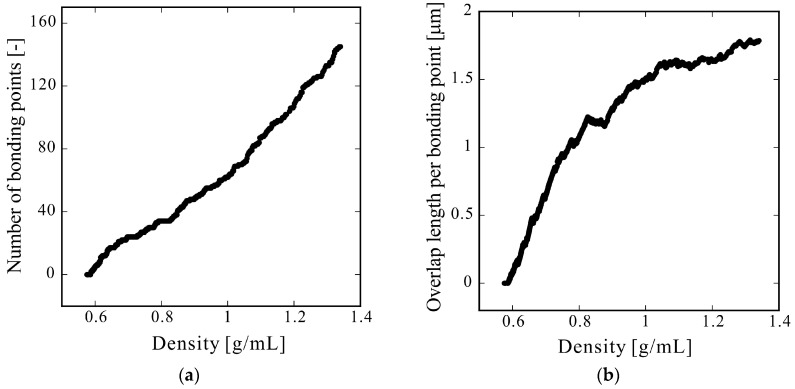
Plots of (**a**) the number of overlap points and (**b**) the average overlap length as functions of density.

The density, number of bonding points, and average overlap length were calculated by varying the compression ratio; the results are plotted against density in Figure 6. The number of bonding points monotonically increased and the average overlap length increased by upward expansion. Some regions were observed in which the average overlap length decreased with increasing density. This behavior was due to a change in the number of bonding points. The overlap length of a newly generated bonding point is extremely small, which decreases the average overlap length. When the analysis area becomes large, this jaggedness will be inconspicuous, and the graph should appear smooth.

**Figure 7:**
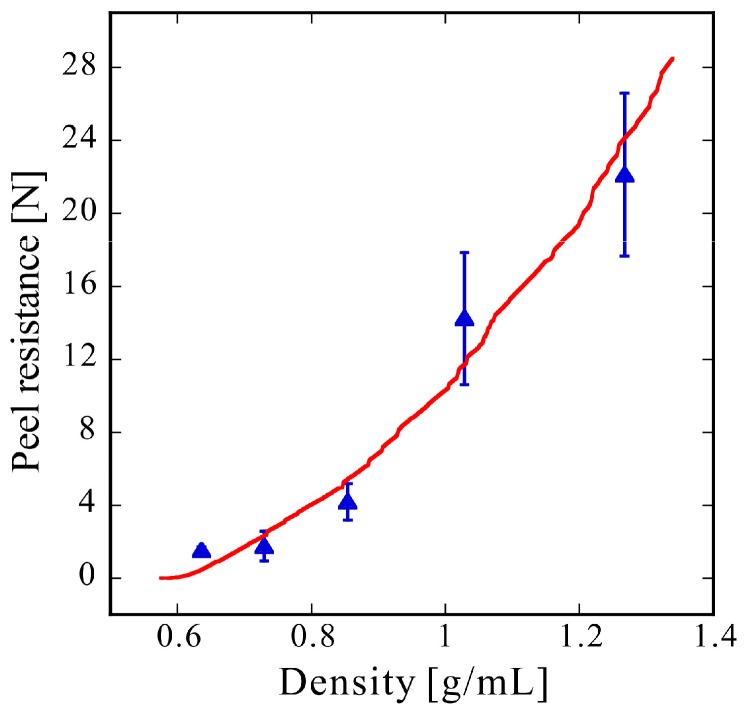
Experimental and simulation results. Blue triangles represent experimental results. Error bars indicate the standard deviation. The red line represents the simulation results obtained using the cross-sectional analysis model developed in this work.

When adhesion comprises of point bonding, the bond strength is calculated as the bond strength per bonding point multiplied by the number of bonding points. Therefore, the experimental results were compared with those obtained by multiplying the number of bonding points in Figure 6a by the bonding distance in Figure 6b. Additionally, because the model calculation was performed in a space of 50 by 100 μm, and the adhesion strength was evaluated from the overlap length, a coefficient was added to match the experimental result. The result of plotting using a coefficient of 0.11 N/μm, which was determined to match the experimental result, is shown in Figure 7. In this figure, triangles represent the average experimental results; here, no individual experimental results are described but the standard deviation is plotted as an error bar. The calculation results are shown in red. These express the increasing peel resistance indicated by the experimental results. The average overlap distance multiplied by the number of adhesion points used for plotting is the sum of the overlap length. This value is the sum of the red line lengths in Figure 5. Thus, if the increase in peel resistance can be evaluated from the sum of the overlap lengths in the cross-sectional analysis, the cross-sectional analysis is potentially a highly effective method for analyzing peel resistance.

## 3. Materials and Methods

### 3.1. Sample Preparation

**Figure 8:**
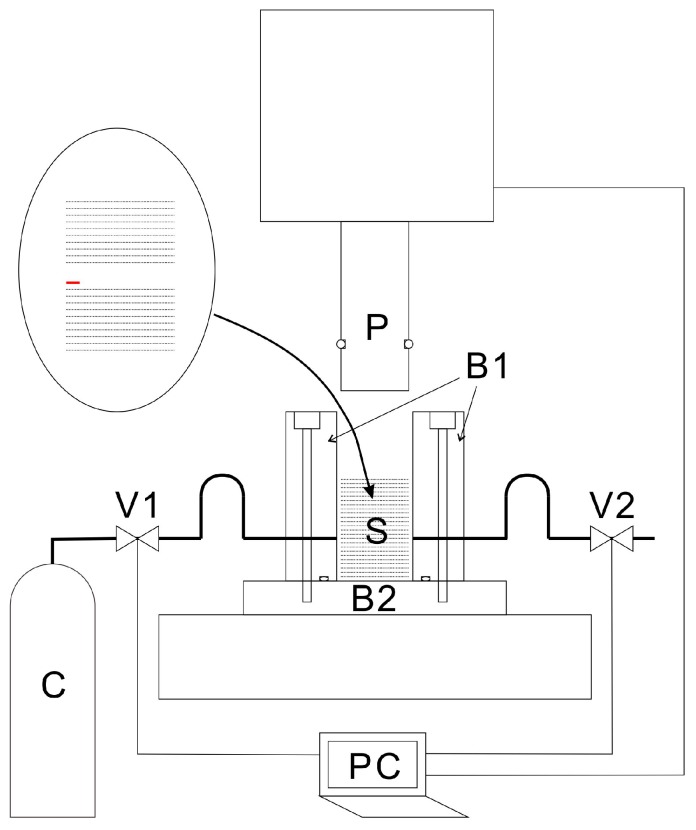
Schematic of a cross-section of the high-pressure vessel. B1: body of the high-pressure vessel; B2: base of the high-pressure vessel; C: CO_2_ cylinder; P: piston; PC: laptop computer; S: sample; V1: intake valve; and V2: exhaust valve.

The CAPC process is detailed elsewhere [17]. Briefly, Figure 8 shows a cross-sectional view of a press unit and a tube-connection diagram. The press machine is a JP-1504 model manufactured by Janome Sewing Machine Co., Ltd (Hachioji, Japan). A piston (outer diameter: 19.5 mm) and pressure vessel (inner diameter: 20.0 mm) were attached to the press machine. Ball valves were connected to the pressure vessel via stainless-steel tubes through which CO_2_ was introduced and exhausted. The valves and piston were controlled with a laptop computer (PC).

The non-woven fabric was identical to that used in a previous study [18], which had an average fiber diameter of 8 μm manufactured by Nippon Nozzle Co., Ltd. (Kobe, Japan), as well as using the melt blown method with TK3 PET pellets manufactured by Bell Polyester Products, Inc (Yamaguchi, Japan). The basis weight of this non-woven fabric was ~30 g/m^2^. The non-woven fabric was cut circularly with an 18 mm-diameter punch.

Firstly, 32 pieces of a non-woven sheet (0.246 g) were loaded into the pressure vessel. The piston was then lowered to a predetermined introduction position, and the air in the pressure vessel was replaced with CO_2_. CO_2_ was subsequently introduced at a given vapor pressure from the gas cylinder, followed by lowering the piston to a predetermined press position and holding for 10 s. Thereafter, CO_2_ was exhausted by opening the exhaust valve, and the sample was removed after raising the piston.

The respective introduction and press-position combinations were 2.2 and 1.6 mm, 2.0 and 1.4 mm, 1.8 and 1.2 mm, 1.6 and 1.0 mm, and 1.4 and 0.8 mm. For each combination, the piston was lowered by 0.6 mm after introducing CO_2_ at the vapor pressure; therefore, the extent of volume reduction in the pressure vessel remained constant. Consequently, the extent of CO_2_ liquefaction also remained constant for all experiments.

As described in a previous report [18], the bulk density was estimated from the thickness of the material center and the total weight of the set of non-woven fabric sheets, while also considering the porosity.

### 3.2. Measurements

Peeling and penetration tests were performed using a Force Tester MCT-2150 from A & D Co., Ltd (Toshima-ku, Japan). For the peeling test, a polyimide tape with a thickness of 30 μm was attached to the edge of the middle layer of the non-woven fabric sheets before loading them into the pressure vessel (Figure 8). Initially, peeling tests were attempted without this polyimide tape by creating a notch at the edge of the middle layer of the porous polymer using a cutter. This notch was slightly peeled off the edges and pinched in a chuck to perform the peeling test. However, the results were irreproducible because the notches were improperly formed at the boundaries between the non-woven fabric sheets. Effectively, the splitting strength of the non-woven fabric was measured in addition to the peel resistance. Inserting a thin film of a network polymer, which is difficult to plasticize with CO_2_, enabled measurement of the boundary between the sheets, thereby allowing reproducible measurement of the peel resistance.

**Figure 9:**
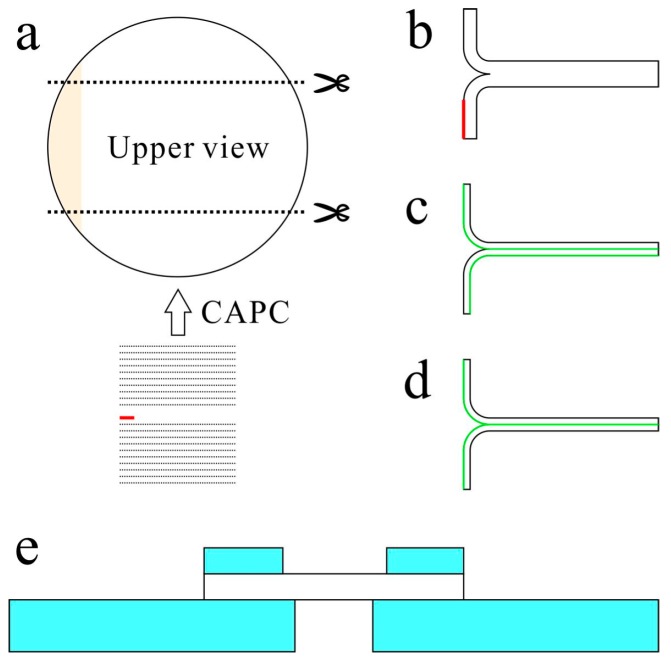
Sample preparation for the peel and penetration tests. (**a**): Cutting point for peel test, (**b**): edge-widened sample for peel test, (**c**): reference sample (one-sided adhesive surface) for peel test, (**d**): reference sample (dual-sided adhesive surface) for peel test, and (**e**): sample sandwiched between steel plates for penetration test.

T-type peel resistance tests were performed by dividing the samples into 16 sheets (Figure 9b). A schematic of the porous PET material with inserted polyimide tape is shown in Figure 9a; the sides were cut at the black dotted lines to prepare samples with widths of ~10 mm. Thereafter, the edges were pulled apart slightly, bent, and set on the chucks to perform a T-type peeling test (Figure 9b). The moving speed of the head in the peeling test was set to 10 mm/min, which was the lowest limit of this equipment. The average width was obtained by measuring at three places, and the peel resistance for a width of 10 mm was calculated by dividing the measured peel resistance by the average width and then multiplying by 10. All measurements were performed thrice, and the values were averaged. For reference, the peel strengths of stationery tape samples, Scotch transparent clear finish tape with a width of 18 mm, bonded with the adhesive surface to the back surface (Figure 9c) and with the adhesive surfaces facing one another (Figure 9d) were measured then converted to resistance values for a width of 10 mm.

For the penetration tests, samples compressed without inserted polyimide tapes were used. Each sample was attached to a steel plate with a hole (⌀6.5 mm) using adhesive, and a second steel plate with a hole (⌀8.5 mm) was then attached on top (Figure 9e). A stainless-steel needle (⌀1 mm) with a hemispherical head (radius: 0.5 mm) was used to puncture the center and measure the penetration resistance. The moving speed of the head in the penetration test was set to 10 mm/min, which was the lowest limit of this equipment. For reference, the penetration resistance of a polyethylene film (thickness: 0.08 mm) and ethylene–vinyl acetate copolymer (EVA) foam (thickness: 5 mm) was also measured. All the measurements were performed thrice, and the values were averaged.

The fiber surface of the central layer was observed with a scanning electron microscope (TM-1000, Hitachi High-Technologies, Minato-ku, Japan) to investigate the bonding state of the sample, which was the surface state of the release surface after the T-type peeling test.

### 3.3. Model Analysis

**Figure 10:**
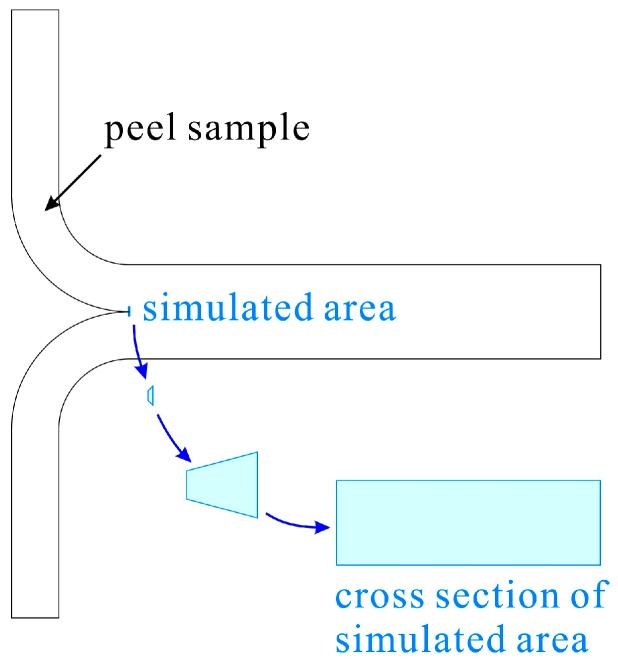
Peel sample and simulated area.

A cross-sectional analysis model was developed to analyze a minute cross-section of the peeled portion in Figure 10. The compression induced an increase in the number of bonding points and the bonding strength per bond point was calculated.

The peel resistance should be analyzed using a width of 10 mm; however, the analysis was performed in a space that was 50 μm in height and 100 μm in width. Additionally, for a cross-section wherein fibers, oriented randomly in a two-dimensional direction, were laminated, aside from the circular fiber cross-section, the elliptical section when the fiber was cut obliquely as well as the rectangle that appears when cuts are made parallel to the fiber direction should be considered. However, the simplest model, which only considered circular cross-sections of fiber, was adopted. Although the model is extremely simplified, it should be sufficient to understand the trend with compression from the same initial state.

**Figure 11:**
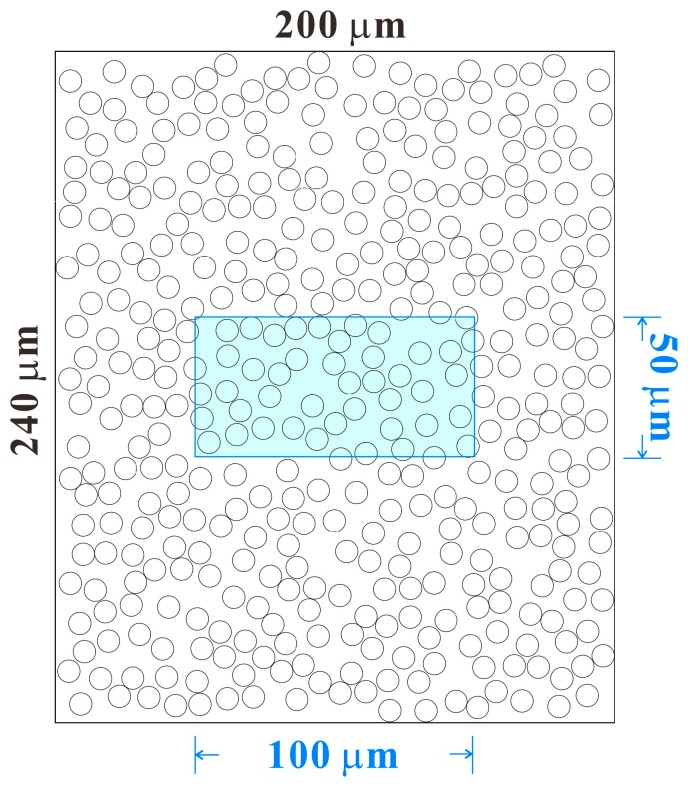
Example of fiber arrangement in the cross-sectional model. The inner frame (outlined in sky blue) is the bonding analysis area.

Firstly, in a space that was 240 μm in height and 200 μm in width, 400 pieces of 8 μm fiber were randomly placed, avoiding overlap (Figure 11). The area of analysis was a space 50 μm in height and 100 μm in width, which was at the center of this space (sky blue rectangle in Figure 11). Compression was performed by moving the circle center positions by applying a constant coefficient (compression rate) toward the center in the longitudinal direction. The definition of the compression ratio in this work was the length after compression divided by the length before compression; therefore, the compression rate is small for high compression and large for low compression. Under fiber compression, the circles that were initially arranged to avoid overlap will overlap.

**Figure 12:**
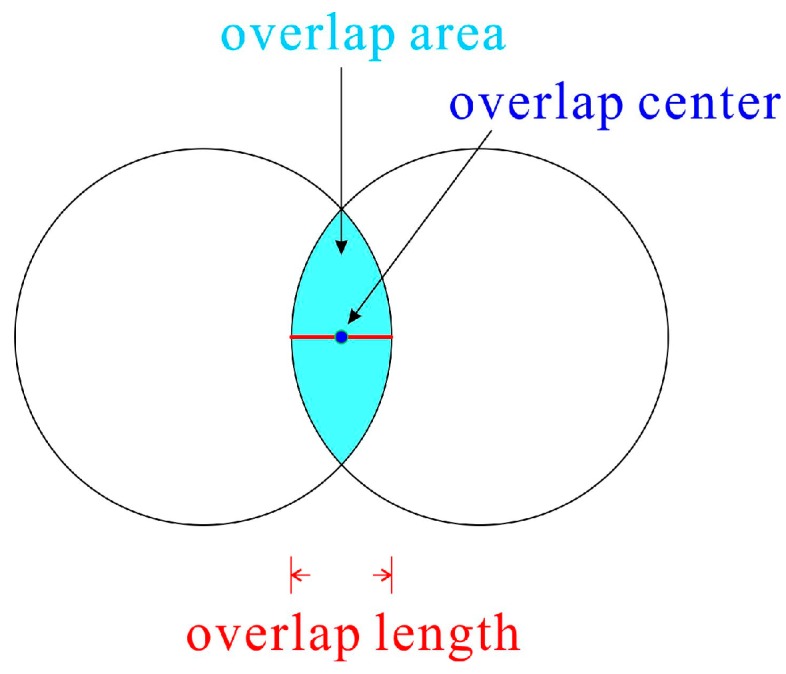
Definitions of the overlap area, overlap center, and overlap length.

Figure 12 shows a state where the circles overlap. The overlap area, overlap center, and overlap length are defined in Figure 12. Three values were calculated for the analysis. The first was the density. For each circle, the area of the region overlapping the rectangular analysis region was summed up, and the area occupied by the fiber in the rectangle was calculated. When the rectangle interior is filled with fibers, its density should be 1.34 g/mL, which is the PET polymer density; therefore, the apparent density of this porous material is (1.34 g/mL times total area of fibers in analysis area) divided by the (analysis area). The density calculated using this method counts the overlapping area twice. However, in actual systems, the overlapping portion deforms so that the polymer moves aside; therefore, both areas must be counted. Additionally, on the boundary of the analysis area, some fibers were forced from the analysis area during deformation; however, fibers outside the analysis area may similarly enter the analysis area under deformation. These effects were considered to offset each other, and no special operation was performed to account for this effect. The number of bonding points, i.e., the overlap centers within the analysis area were then counted. Finally, the compression strength of a bonding point was evaluated as the overlap length; i.e., the average overlap length of the bonding points where the overlap center was in the analysis area was calculated and analyzed, because this overlap length correlates with the bonding strength of the bonding point.

## 4. Conclusions

Herein, the relation between the processing conditions and the peel and penetration resistance of porous PET materials produced by CAPC was clarified for the first time. The produced samples could be cut or bent, and industrial processing is possible. The penetration resistance gradually increased with compression. However, the peel resistance rapidly increased with the compression, exhibiting an adhesive strength which is adequate for industrial applications. Evaluating the adhesive strength using a cross-sectional analysis model revealed that the increasing tendency of adhesive strength was well expressed, suggesting that the cross-sectional analysis model is efficient. Herein, only analysis using an 8 μm diameter PET fiber with a circular cross-section was discussed; however, if the analysis can be adapted to make it independent of the fiber type, fiber diameter, and cross-sectional shape, the cross-sectional analysis model may be a powerful analysis tool for CAPC materials.

The CAPC-processed porous PET material may be suitable for protective purposes because it exhibits high penetration resistance resulting from the lamination of fibrous sheets. Given that the through-holes are open, the material is unlikely to steam because water vapor can be easily released. Thus, the material should be suitable for use in supportive clothing such as medical corsets as well as protective athletic gear.
